# Genetic variability within *Triatoma sanguisuga* complex in North America and the redescription of *Triatoma ambigua* (Neiva 1911), with specimens from Florida, USA

**DOI:** 10.21203/rs.3.rs-9955037/v1

**Published:** 2026-06-11

**Authors:** Norman L. Beatty, Sebastian Pita, Bruno A. Sansoni-Ruidίaz, Sergio Mendez-Cardona, Bernardo Moreno Peniche, Chanakya R. Bhosale, Susan E. Halbert, Catherine E. Nance, Paul E. Skelley, Francisco Panzera, Eva Nováková, Samantha M. Wisely, Rachel Curtis-Robles, Mainul Hasan Sarker, Juan David Ramírez, Gabriel L. Hamer, Sarah A. Hamer

**Affiliations:** 1 Division of Infectious Diseases and Global Medicine, University of Florida College of Medicine, Gainesville, FL USA; 2 Emerging Pathogens Institute, University of Florida, Gainesville, FL USA; 3 Sección Genética Evolutiva, Facultad de Ciencias, Universidad de la República, Montevideo, Uruguay; 4 Florida Medical Entomology Laboratory, Vero Beach, Florida, USA; 5 University of California, Berkeley, CA, USA; 6 Dr. Kiran C. Patel College of Osteopathic Medicine, Nova Southeastern University, Fort Lauderdale, Florida, United States of America; 7 Florida Department of Agricultural and Consumer Services, Division of Plant Industry Gainesville, FL, USA; 8 Faculty of Science, University of South Bohemia in Ceske Budejovice, Czech Republic; 9 Department of Wildlife Ecology and Conservation, University of Florida, Gainesville, FL, USA; 10 Department of Veterinary Integrative Biomedical Sciences, Texas A&M University, College Station, Texas, USA; 11 Center for Global Health and Inter-Disciplinary Research, USF Genomics Program, Department of Global, Environmental and Genomic Health Sciences, College of Public Health, University of South Florida, Tampa, FL, USA; 12 Centro de Investigaciones en Microbiología y Biotecnología-UR (CIMIBIUR), School of Sciences and Engineering, Universidad del Rosario, Bogotá, Colombia; 13 Department of Entomology, Texas A&M University, College Station, Texas, USA

**Keywords:** Triatominae, *Triatoma ambigua*, *Triatoma sanguisuga*, kissing bug, Chagas disease

## Abstract

*Triatoma sanguisuga* is the most widely distributed triatomine vector in North America, occurring in at least 23 U.S. states across and capable of transmitting *Trypanosoma cruzi* to humans. Historical morphological variability led to the description of several subspecies, but their taxonomic validity has remained unresolved. We integrated molecular (cytochrome b, n=58; ITS-2, n=23) and morphometric (18 characters, n=82 specimens) data to reassess species boundaries within *T. sanguisuga* sensu lato. Four species-delimitation methods (GMYC, ABGD, mPTP, hierBAPS) consistently identified four divergent mitochondrial lineages with K2P distances of 4.5–8.8%. Florida specimens formed a distinct, strongly supported clade (Group 1; K2P >7.5% from other groups) corresponding to *T. sanguisuga* var. *ambigua* (Neiva, 1911). Linear discriminant analysis achieved 100% classification accuracy between Florida and non-Florida morphotypes, with synthlipsis width providing the strongest diagnostic character (AUC >0.96). Based on concordant genetic and morphological evidence, we revalidate *Triatoma ambigua* (Neiva, 1911) stat. nov. as a distinct species within a newly defined *T. sanguisuga* complex and designate a lectotype from the Instituto Oswaldo Cruz collection. Sampling focused primarily on Florida; additional sampling in adjacent southeastern states is needed to characterize geographic boundaries. These findings have implications for Chagas disease vector surveillance in the southeastern United States.

## Introduction

Chagas disease, caused by the protozoan parasite *Trypanosoma cruzi* (Chagas, 1909), is a neglected tropical disease endemic to North, Central and South America, with an estimated 6–8 million people infected worldwide ^1,2^. Transmission occurs primarily through contact with the feces of infected triatomine bugs, although congenital transmission, blood transfusion, organ transplantation and ingestion of contaminated food are also recognized routes ^3^. The infection typically progresses from an acute phase, often asymptomatic or associated with nonspecific signs, to a chronic phase that may remain clinically silent for decades. Approximately 30% of chronically infected individuals develop Chagas cardiomyopathy, which can lead to heart failure, arrhythmias and sudden cardiac death, resulting in considerable morbidity and mortality ^4^. Despite this burden, Chagas disease remains markedly underdiagnosed and undertreated.

In the United States (US), Chagas disease is recognized as an endemic zoonosis ^1^. While most infections have been acquired in Latin American countries, an estimated 300,000–347,000 people living in the US are chronically infected with *T. cruzi*, and both imported and autochthonous cases have been documented ^1,3,4^. Autochthonous vector-borne transmission has been reported from multiple southern states, including Texas and other regions along the US–Mexico border, where infected triatomines, competent wildlife reservoirs and human exposure overlap ^5,6^. At least 11 triatomine species are known from the US, many of which harbor *T. cruzi*, indicating that ecological conditions for transmission are present in several areas ^1,5^. However, fewer than 1% of infected individuals in the US are thought to receive a diagnosis, reflecting low clinical awareness and limited routine screening ^4^. Robust data on the distribution, infection status and taxonomy of North American triatomines are therefore fundamental for risk assessment and surveillance ^7^.

Florida is a key setting for understanding the eco-epidemiology of Chagas disease in the United States. The state has evidence of sylvatic *T. cruzi* transmission cycles and documented invasion of human dwelling by triatomine vectors , supporting its relevance for understanding potential local exposure risk ^7^. Recent field investigations in Florida documented *T. cruzi*-infected triatomines, predominantly *Triatoma sanguisuga* (LeConte, 1855), in and around human dwellings across 23 counties, indicating substantial domestic and peridomestic human–vector contact ^8^. *T. sanguisuga* is the principal triatomine reported from Florida and has historically been recorded broadly across the state, including incursions into homes and feeding on humans and domestic animals ^8,9^. In a 2025 Florida dataset, 29.5% of tested *T. sanguisuga* were positive for *T. cruzi*, indicating local transmission potential, although no locally acquired human cases have been officially reported from Florida to date ^7,8^. In addition, raccoons and opossums show frequent infection and occur in peridomestic habitats, supporting active transmission cycles near human habitations ^10,11^.

Beyond Florida, *T. sanguisuga* is among the most widely distributed triatomines in North America. It occurs from the southern Great Plains through the southeastern and Mid-Atlantic states, with records from Texas and Louisiana to Florida and as far north as Delaware ^1,6,12,13^. Across this range, *T. sanguisuga* occupies diverse ecological regions, including the Great Plains, eastern temperate forests and subtropical habitats of the Gulf Coast and southern Florida ^14^. Reported *T. cruzi* infection prevalences in *T. sanguisuga* are highly variable, with approximate rates of 30% in Florida, >40% in parts of Texas and >60% in Louisiana ^8,15–17^. Blood-meal analyses indicate a broad host range, including reptiles, birds, amphibians and multiple mammalian species, humans among them, consistent with an opportunistic feeding strategy influenced by local habitat and host availability ^18–22^. Indoor-collected *T. sanguisuga* often show higher frequencies of human and domestic animal blood, whereas outdoor-collected bugs near homes in Florida more commonly feed on wild mammals and amphibians ^8^. This combination of broad geographic distribution, ecological plasticity, high *T. cruzi* infection prevalences and frequent peridomestic incursion underscores the importance of *T. sanguisuga* as a Chagas disease vector in North America.

As in many triatomine taxa, however, the taxonomy and intraspecific structure of *T. sanguisuga* remain incompletely resolved. The species exhibits considerable variation in external morphology, including body size and connexivum coloration ^23^. Early in the twentieth century, this variation led Neiva (1911) ^24^ to describe *T. sanguisuga* var. *ambigua* from specimens collected in “Narrows-Florida”, a locality generally associated with the present-day Indian River Lagoon region near Jupiter, Florida. Subsequent work recognized *T. s. texana* in south Texas and *T. s. ambigua* in Florida ^25,26^, and additional morphotypes such as “*Conorhinus lateralis*” and “*Triatoma pintoi*” have been mentioned. *T. sanguisuga* also closely resembles its sister species *T. indictiva*, and discrimination between them, especially in nymphal stages and in zones of sympatry, can be challenging ^6,27^. Lent and Wygodzinsky (1979) treated these subspecies as invalid and interpreted the observed variation as clinal, but subsequent molecular and morphometric evidence has suggested a more complex scenario ^23^.

Genetic studies indicate substantial diversity within *T. sanguisuga*. Along the Georgia barrier islands, mtDNA COII data showed strong population structuring, with no shared haplotypes between islands and evidence of limited dispersal/gene flow ^28^. In Louisiana, mtDNA analyses (*cytochrome b* and 16S) identified deeply divergent haplotype groups within a single local population, with divergence levels interpreted as potentially consistent with infraspecific subdivision ^29^. Broader systematics work using nuclear ITS-2 and morphometrics across North/Central American *Triatoma* species supported revision of species-complex relationships but did not fully resolve all taxonomic boundaries ^30^. Recent phylogenomic analyses using ultraconserved elements have improved higher-level relationships within *Triatominae/Triatomini*, while highlighting remaining uncertainty in parts of the tree ^31^. The first high-quality *T. sanguisuga* genome, generated from a Delaware specimen, now provides a foundation for testing population divergence and reproductive isolation hypotheses with genome-scale data ^14^.

Taken together, the marked morphological variability, deep but incompletely resolved genetic divergence and broad ecological amplitude of *T. sanguisuga* suggest that it may represent a complex of closely related taxa rather than a single homogeneous species. This possibility has important implications for Chagas disease surveillance and risk assessment, as distinct lineages may differ in vector competence, host associations and behavior. Florida is particularly relevant in this regard, as it is both the type of region for Neiva’s *ambigua* form and a state where *T. sanguisuga* is a common, *T. cruzi*-infected peridomestic vector species. In this study, we integrate molecular and morphometric data from *T. sanguisuga* sensu lato in Florida to reassess the taxonomic status of Neiva’s *ambigua* form. Our analyses support the recognition of *Triatoma ambigua* (Neiva, 1911) as a distinct species within the *T. sanguisuga* complex and refine understanding of vector diversity relevant to Chagas disease risk in the southeastern United States.

Given the substantial genetic and morphological diversity documented within *T. sanguisuga*, we here refer to “*T. sanguisuga* complex” to encompass *T. sanguisuga* sensu stricto, *T. ambigua* (as revalidated herein), and potentially additional undescribed lineages identified by molecular species delimitation. This usage follows integrative taxonomic practice in other triatomine groups (e.g., *T. brasiliensis* complex) and does not imply formal recognition under the species-complex definitions of Lent and Wygodzinsky (1979) ^23^.

## Results

### Genetic Diversity and Population Structure

A total of 58 new *cytb* sequences (453 bp) from *Triatoma sanguisuga* specimens were analyzed alongside 96 sequences retrieved from GenBank. The cytb sequence from the recently published Delaware genome ^14^ was not considet at the time for species delimitation analyses but was subsequently examined to determine its lineage assignment. A total of 113 distinct haplotypes were identified and designated H_1 through H_113. The geographic distribution of specimens included in the cytb analysis is shown in Supplementary Figure S1, revealing clear spatial clustering of GMYC-defined groups. The most frequently observed haplotype, designated H_31, was found in specimens from Texas, Tennessee, Virginia, and Louisiana. The distribution of haplotypes exhibited clear geographic organization, with most haplotypes clustering within the sample regions. Notably, all Group 1 haplotypes were detected exclusively in Florida specimens; however, this pattern may reflect our sampling focus rather than a true distributional boundary, as adjacent states (e.g., Georgia, Alabama) were underrepresented in our dataset. Samples from Texas exhibited high haplotype diversity. H_58 was detected in both Tennessee and Virginia, which may indicate genetic continuity between populations in these adjacent regions. Similarly, H_113 appeared in Texas, Tennessee, West Virginia, and Iberville (Louisiana), making it the most widely distributed haplotype in the dataset. The broad presence of H_113 suggests that it could be an ancestral variant maintained across multiple populations. Worth mentioning that the mitochondrial haplotipe from the genome assembly was one mutation away to H_55.

**Figure 1:**
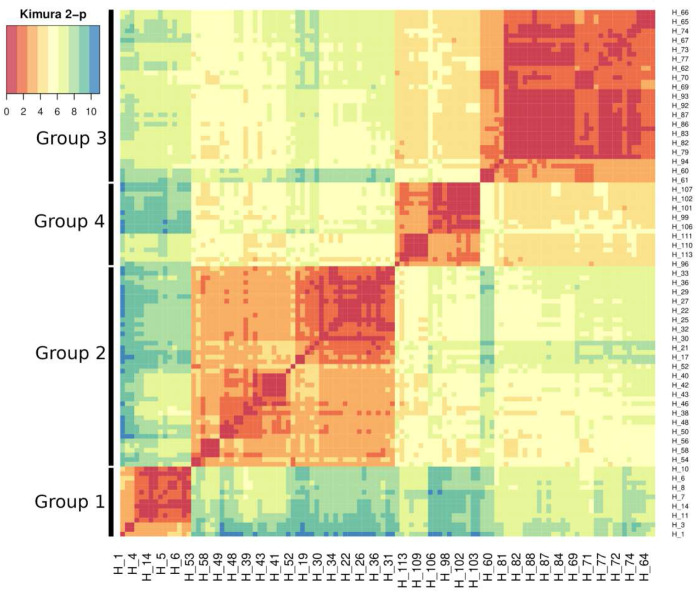
Genetic pairwise distances among *Triatoma sanguisuga CYTB* haplotypes. Heatmap of Kimura 2-parameter (K2-p) pairwise distances between 113 mitochondrial cytb haplotypes (H_1–H_113) from *T. sanguisuga* across the southeastern United States. Haplotypes are arranged by the four GMYC-defined lineages (Group 1–4) indicated along the axes. Color intensity indicates genetic distance (light = low, dark = high). Diagonal blocks show within-group distances; off-diagonal blocks show between-group distances. Group 1 (Specimens from Florida in this study) shows the greatest divergence from the other three groups (>7.65% K2-p), while distances among Groups 2, 3 and 4 range from 4.50% to 6.54%. High within-group distances in Groups 1 and 2 indicate marked haplotype diversity; Groups 3 and 4 are more homogeneous.

Species delimitation was inferred using four single locus approaches. All four were highly coincidental, identifying four principal lineages within *T. sanguisuga*. Two of them had slight differences: mPTP subdivided group 4 in several putative species; hierBAPS split group 2 in two putative species. Since GMYC have the same results as ABGD, and coincides in part with mPTP and hierBAPS, we are basing our further analysis on this grouping’s results. The *cytb* dataset contained 116 segregating sites (25.61%) and a high haplotype diversity (Hd = 0.980) but a low nucleotide diversity (π = 0.046) (Table 1.). Neutrality tests supported a lack of significant departure from neutrality for the entire dataset (Tajima’s D = 0.121, p = 0.903; R2 = 0.089, p = 0.608). However, Group 1, defined by GMYC, exhibited a significant R2 value (0.084, p = 0.036), suggesting possible demographic expansion or selection. Genetic pairwise distance between GMYC defined groups is depicted in table 2. Intergroup distances ranged from 5.0% to 8.8% (K2-p). Briefly, the Group 1 (Specimens from Florida) presented the largest distance with the other three groups (larger than 7.65%). Whilst among the other three groups, distances varied from 4.50% to 6.54%. Genetic pairwise distance measured between haplotypes is depicted as a heatmap in Figure 1.

For the nuclear marker ITS-2 (830 bp), a total of 11 different haplotypes were detected among the 23 samples analyzed and codified as ITS2-1 to ITS2-11 (Supplementary Table 1). The distribution of haplotypes exhibits clear geographic organization, with some haplotypes restricted to specific regions and others showing a more dispersed presence. Haplotype ITS2-1 was the most represented in the sample, with eight individuals, all from Texas. In addition, haplotypes ITS2-2, ITS2-3, and ITS2-4 were also recorded in this state, none of which appear in other regions. Haplotypes ITS2-5 and ITS2-6 were found in Mississippi and Alabama. ITS2-5 appeared in both states, while ITS2-6 was detected only in Mississippi. In Virginia and Tennessee, haplotypes ITS2-7 and ITS2-8 were recorded, respectively, each represented by a single individual. In Florida, three distinct haplotypes were identified: ITS2-9, ITS2-10, and ITS2-11. ITS2-9 was the most frequent within the state, with four individuals distributed across different locations, while ITS2-10 and ITS2-11 appeared in only one sample each. Texas and Florida were the only states that exhibited multiple haplotypes not shared with other regions.

### Phylogenetic Relationships

**Figure 2:**
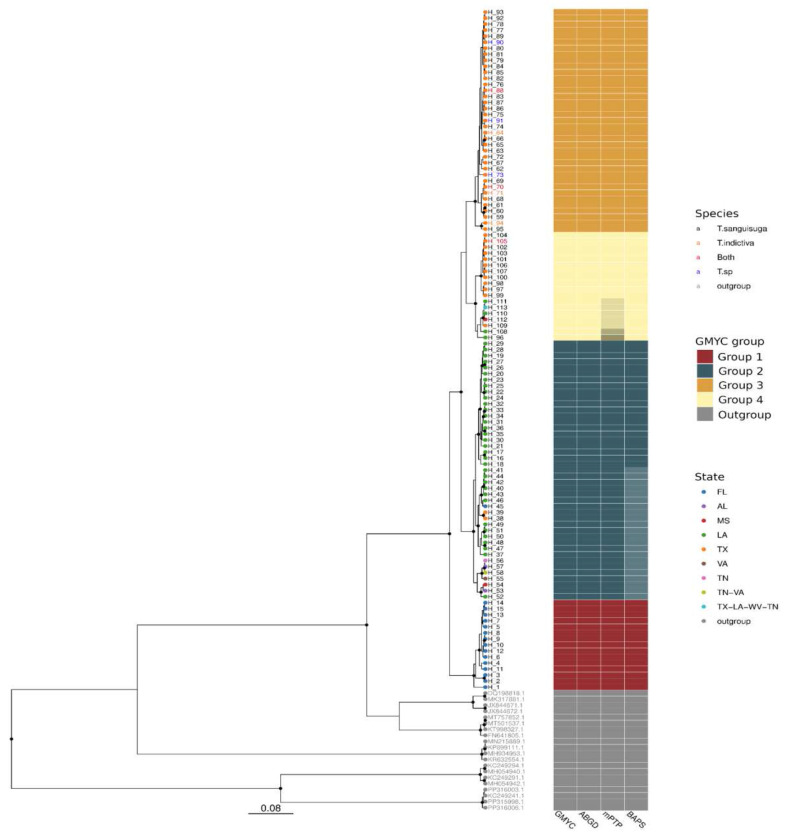
Bayesian phylogeny and single-locus species-delimitation results for *Triatoma sanguisuga CYTB* haplotypes. Bayesian inference tree of the mitochondrial cytb fragment showing relationships among *T. sanguisuga* Isensu lato and related taxa across the southeastern United States. Black circles on nodes indicate well-supported clades (posterior probability > 0.95). Tip labels correspond to haplotypes (H_1–H_113), with colored tip symbols indicating the U.S. state of origin (see legend) and colored tip text marking sequences deposited in GenBank as species other than *T. sanguisuga* (e.g. *T. indictiva*, *Triatoma* sp.), highlighting potential misidentifications or taxonomic ambiguity. The four GMYC-defined groups (Groups 1–4) form the main clades of the tree: Group 1 was comprised exclusively of Florida specimens that were strongly divergent from all others; Group 2 included mainly Louisiana specimens plus a few from more northern states; Group 3 included only specimens from Texas and contained sequences labelled as *T. sanguisuga, T. indictiva* and *Triatoma sp.*; Group 4 was composed primarily of Texas and Louisiana specimens. The right-hand heatmap summarizes congruent species-delimitation assignments from GMYC, ABGD, mPTP and hierBAPS, with each color representing a distinct putative species or outgroup lineage. The scale bar indicates substitutions per site.

Based on the *cytb* marker, four distinct genetic groups were identified using GMYC. The Bayesian phylogenetic tree generated with BEAST showed high support (posterior probability > 0.95) for four main clades (Figure 2). Group 1: Composed exclusively of Florida specimens in our sampling, showing significant genetic divergence from the other groups (average K2-p distance > 7.5%). This group exhibited the highest haplotype diversity (Hd = 1) but was represented by the smallest number of specimens (n = 11). Group 2: Predominantly from Louisiana, with a few specimens from more northern states (e.g., Tennessee, Virginia). This group displayed the highest nucleotide diversity (π = 0.023) and strong internal structuring, as evidenced by HierBAPS subgroups. Group 3: Composed of specimens from Texas in our dataset, including samples identified as *T. sanguisuga, T. indictiva* and *Triatoma* sp. – indicating potential misidentification or taxonomic ambiguity. This group displayed low nucleotide diversity (π = 0.016) and moderate haplotype diversity (Hd = 0.987). Group 4: Consisted primarily of specimens from Texas and Louisiana but showed weaker support in ML and MP phylogenies. Some haplotypes (e.g., H34, H104) appeared as outliers in phylogenetic and haplotype network analyses.

**Figure 3:**
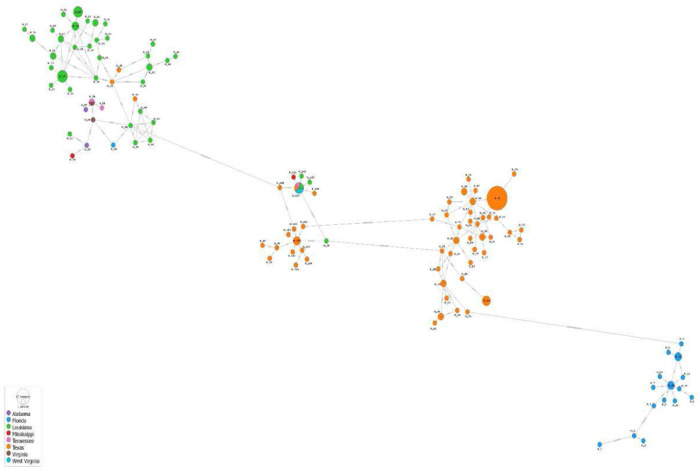
Haplotype network (minimum spanning tree) of partial *cytb* sequences from *T. sanguisuga* sensu lato. Node size is proportional to haplotype frequency; hash marks along edges indicate the number of mutational steps between haplotypes. Node colors indicate U.S. state of origin (see legend). The four GMYC-defined groups are indicated: Group 1 (Florida, green), Group 2 (Louisiana/central states, blue), Group 3 (Texas, orange), and Group 4 (Texas/Louisiana, light orange). Group 1 is separated from the nearest group by 22 mutational steps, indicating substantial genetic divergence.

The haplotype network (Figure 3) revealed clear separations between the four groups, with Group 1 showing the most significant divergence (22 mutational steps from the nearest group). Groups 3 and 4 were more closely related, with 13 mutational steps between their central haplotypes.

**Figure 4:**
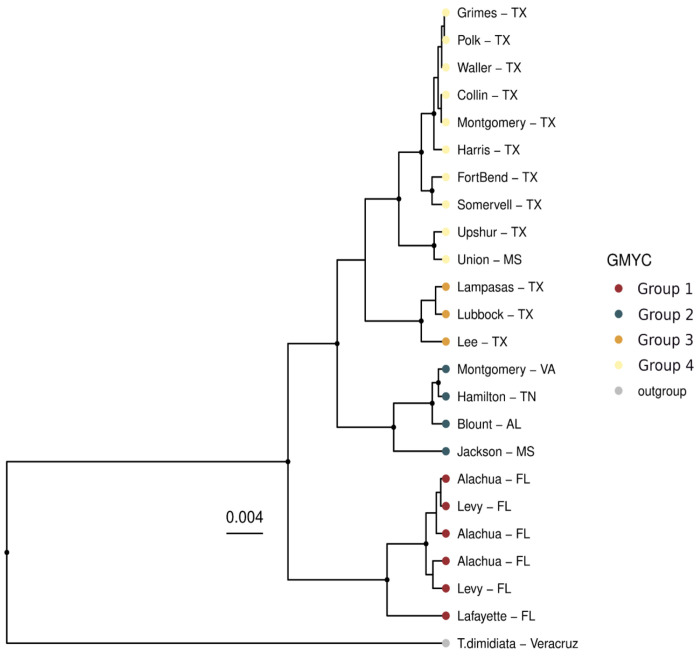
Partitioned bayesian inference phylogenetic tree using *cytb* gene fragment and ITS-2 marker. Black dots on the nodes represent posterior probability greater than 0.95. Colors of the dots represent the GMYC group of the samples determined in *cytb* analysis. The US States are abbreviated as FL: Florida, MS: Mississippi, AL: Alabama, TN: Tennessee, VA: Virginia, TX: Texas. The outgroup is *T. dimidiata* from Veracruz State, Mexico.

The partitioned phylogenetic tree constructed using both: *cytb* (mitochondrial) and ITS-2 (nuclear) markers revealed a clear separation into two main lineages (Figure 4). On one hand, some samples from Florida (Lafayette, Levy, and Alachua Counties) formed a distinct and well-supported clade, which was completely separated from all other populations included in the analysis. This pattern was also observed in the tree based solely on *cytb* (Figure 3), and it remained consistent in the analysis incorporating nuclear data. On the other hand, the second major lineage, encompassing the remaining samples, exhibited a further division into two primary clades: a clade comprising individuals from Texas, representing the westernmost populations in the dataset, and a clade that included samples from Mississippi, Alabama, Tennessee, and Virginia, corresponding to a central-southern region of the United States. This phylogenetic structure suggests a clear differentiation between the western populations (Texas) and the central-southern populations (Mississippi, Alabama, Tennessee, and Virginia), both of which are phylogenetically distinct from the Florida lineage, which remains as an independent and well-supported group.

**Figure 5.**
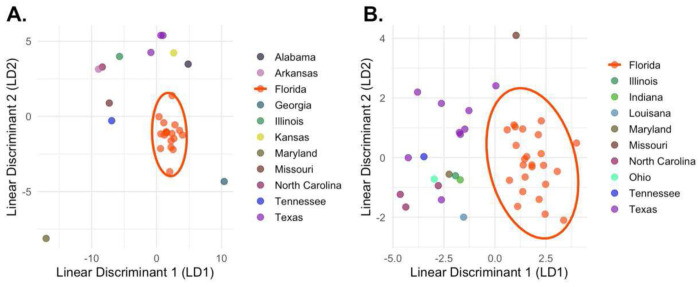
Linear Discriminant Analysis (LDA). Scores for morphological characters of specimens grouped by state, shown separately for males (left) and females (right). Points represent individual samples colored by state. A 95% confidence ellipse is drawn around the group of specimens collected from Florida to highlight its morphological clustering.

Initial exploratory analyses using linear discriminant analysis (LDA) based on geographic states revealed clear clustering of specimens from Florida, suggesting potential morphological differentiation within the sample. To further quantify this, one-way ANOVAs were conducted to test the effect of state on LD1 scores separately for males and females from either Florida or non-Florida specimens (Figure 5). For males, there was a highly significant effect of state on LD1 (F(10, 19) = 77.91, p < 0.001), indicating pronounced morphological differentiation among states. Similarly, females showed a significant effect of state on LD1 (F(9, 33) = 22.76, p < 0.001), confirming significant morphological variation across geographic groups. For males, the first two linear discriminants (LD1 and LD2) explained 86.7% of the between-group variance, with LD1 accounting for 65.4% and LD2 for 21.3%. In females, these two axes captured 80.2% of the between-group variance (LD1: 68.0%, LD2: 12.2%), demonstrating that the primary axes of variation effectively summarize morphological differences among states.

**Figure 7.**
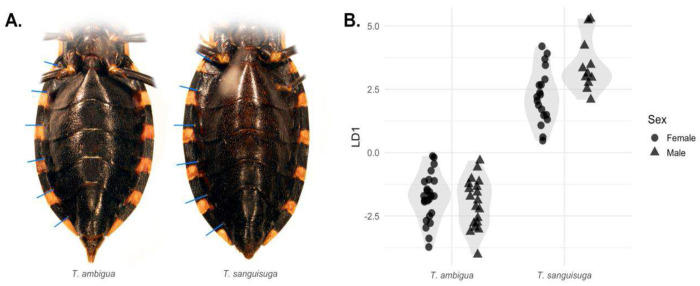
A. Distinct ventral connexivum patterns in the two morphotypes observed. (Left) Morphotype of a sample from Florida (*T. ambigua*) showing apical pale areas consistently reaching the spiracle across all segments. (Right) Morphotype of a sample not from Florida (*T. sanguisuga*) with apical pale areas that do not reach or inconsistently reach the spiracle. **B.** Linear discriminant analysis (LDA) plot demonstrating clear morphological separation between Florida (*T. ambigua*) and non-Florida (*T. sanguisuga*) morphotypes based on selected characters. The LDA model shows strong classification accuracy supporting distinct morphotype groupings.

Closer examination of specimens from Florida revealed a distinctive pattern in the connexivum (Figure 7A). In all specimens from Florida, and one specimen from Georgia, the apical light-colored areas of the connexivum consistently extended to the level of the spiracle across all segments. This contrasted with specimens from other states, where these pale areas either failed to reach the spiracle or ded so only sporadically across segments. Notably, this latter pattern was evident in the lectotype material of *T. sanguisuga* deposited in the Hemimetabola Collection of the Museum für Naturkunde, Berlin ^32^.

Following this morphological distinction, a second LDA was conducted using these morphotype groupings to evaluate the discriminatory power of the selected characters (Figure 7B). The model demonstrated high classification accuracy in distinguishing *T. ambigua* (predominantly found in Florida) from *T. sanguisuga*, confirming that these two taxa are morphologically distinct.

One-way ANOVA was conducted to assess the effect of species grouping on LD1 scores. For females, the analysis revealed a highly significant difference between the Florida (*T. ambigua*) and non-Florida (*T. sanguisuga*) taxa (F_(1, 41)_ = 169.9, p < 0.001), indicating strong morphological differentiation. Similarly, when analyzing the combined dataset of both sexes, the effect of species on LD1 was also highly significant (F_(1, 71)_ = 296.3, p < 0.001), further confirming the robustness of the distinction between these two groups across the sampled individuals.

Confusion matrices constructed from the LDA predictions demonstrated perfect classification accuracy for both males and females. For males, all *T. ambigua* (n = 19) and *T. sanguisuga* (n = 11) individuals were correctly classified, yielding an overall accuracy of 100% (Kappa = 1). Similarly, females were classified with complete accuracy, correctly assigning 24 *T. ambigua* and 19 *T. sanguisuga* specimens, confirming the robustness of the morphotype-based LDA model. To complement these findings, random forest classification models were also implemented. The random forest trained on the full male dataset similarly achieved perfect accuracy (100%) in discriminating species morphotypes, consistent with the LDA results. However, when evaluated on the independent male test subset (from an 80:20 train-test split), classification accuracy decreased to 80%, reflecting a modest reduction in predictive performance on unseen data, but still indicating strong discriminatory power (Kappa = 0.615). For females, the random forest model trained on the complete dataset also achieved perfect accuracy (100%), and importantly, classification on the female test subset maintained 100% accuracy, demonstrating robust predictive performance in females even on unseen data. Examination of the LDA loadings revealed that the primary axis of morphological differentiation between *T. ambigua* and *T. sanguisuga* was predominantly influenced by synthlipsis (intraocular distance) width (Figure 13). Specifically, *T. ambigua* specimens consistently display a narrower interocular distance, a trait that robustly distinguishes them from *T. sanguisuga* across both males and females.

Receiver Operating Characteristic (ROC) curve analysis demonstrated excellent diagnostic performance for the synthlipsis measurement. For females (AUC = 0.997, Youden’s J = 0.95), an optimal cutoff of 0.875 mm maximized discrimination between species. Individuals with synthlipsis values below 0.875 mm were more likely to belong to *T. ambigua*, whereas those with values equal to or above this threshold were classified as *T. sanguisuga*. For males (AUC = 0.963, Youden’s J = 0.86), a slightly lower cutoff of 0.82 mm was optimal, with values below this threshold indicating *T. ambigua*, and values equal to or above it indicating *T. sanguisuga*.

### *Triatoma ambigua* Neiva, 1911, stat. nov.

*Triatoma sanguisuga ambigua* Neiva, 1911, p. 422. Usinger, 1944, p.68.

### Type Material

#### Lectotype.

**Figure 11.**
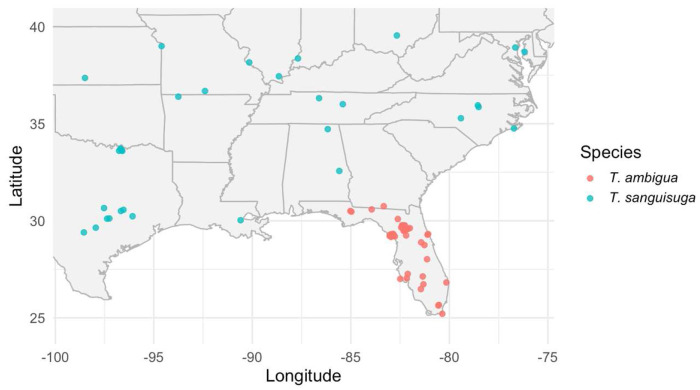
Geographic distribution of sampled specimens included in the morphological analysis. Points indicate morphologically identified *T. ambigua* and *T. sanguisuga* specimens examined in this study; this map does not represent the subset used for molecular analyses.

To stabilize the application of the name *Triatoma ambigua* (Neiva, 1911) (originally described as *Triatoma sanguisuga* var. *ambigua*), we designate as lectotype the historical specimen deposited in the Coleção Entomológica do Instituto Oswaldo Cruz (CEIOC, Rio de Janeiro, Brazil) under catalogue number CEIOC 10020 (previous register CCP1089). The original Instituto Oswaldo Cruz label associated with this specimen (Figure 11A) bears the register number “1089” and identifies the taxon as “*T. sanguisuga var. ambigua*”, with collector P.R. Uhler (“S. Fla, EUA”) and determination by A. Neiva. The specimen, an adult preserved in a sealed glass tube (Figure 11B), originates from Florida, USA (Lake City) and is part of Neiva’s working collection; it is therefore consistent with the concept and type region cited in the original description of *ambigua*. A complete list of submitted specimens assessed at CEIOC around the timeframe of interest is found in Supplementary Table 2.

#### Paralectotype.

One additional adult specimen labelled *T. sanguisuga* var*. ambigua* and preserved in CEIOC under catalogue number CEIOC 10086 (previous register CCP1162) is here designated as paralectotype. This specimen was collected in Florida, USA (exact locality not recorded) in 1937 by A. Packchanian, bears Neiva’s taxon name on the label, and was offered to CEIOC by Packchanian. Although collected after Neiva’s original publication, this specimen has historically been treated as part of the *ambigua* material in Neiva’s collection and is designated as paralectotype following the present lectotype selection.

### Differential diagnosis

*Triatoma ambigua* was first described as a subspecies of *T. sanguisuga* based on its smaller overall size and distribution (Neiva, 1911; Usinger, 1944). Although our observations confirm that *T. ambigua* is generally smaller, total length is variable and not dependable for diagnosis. On closer examination, *T. ambigua* differs from *T. sanguisuga* in the extent of the pale areas on the connexivum and the interocular distance (synthlipsis). In *T. ambigua*, the pale areas of the connexivum consistently extend ventrally to the level of the spiracle across all segments, whereas in *T. sanguisuga s.l.*, the pale areas do not reach or only partially reach the spiracle in some segments (Figure 7A, Figure 13B). The synthlipsis is narrower in *T. ambigua* (<0.875 mm in females, <0.82 mm in males) compared with the broader synthlipsis of *T. sanguisuga* (≥0.875 mm in females, ≥0.82 mm in males) (Figure 13).

### Description

Overall color from dark brown to black, with orange-red markings on neck, pronotum, hemelytra and connexivum. Pilosity inconspicuous. Total length of male 16.5–20.8 mm, of female 16.5–22.2 mm; maximum width of pronotum of male and female 3.8–5.02 mm, of abdomen of male 3.74–4.96 mm, of female 4.05–5.02 mm (Table 3). Head granulose, uniformly dark, approximately 1.77 times as long as wide across the eyes in males (1:0.57) and 1.84 times in females (1:0.54); about the same length as the pronotum in both sexes (1:0.99). Postocular region distinctly rounded in dorsal view; anteocular region about 2.65 times as long as postocular region in both sexes (1:0.38). Eyes in lateral view attaining or slightly surpassing the level of the ventral surface of the head, remote from the dorsal surface. Ratio of width of eye to synthlipsis about 1:1.64 in males and 1:1.79 in females. Antenniferous tubercles situated on the posterior third of the anteocular region. First antennal segment falling slightly short of apex of clypeus; second segment with short hairs only; ratio of antennal segments in males 1:3.93:0.39:0.44, in females 1:4.07:0.41:0.58. Rostrum bearing sparse, very short setae, with a distinct cluster of slightly longer setae near the apex of the third segment; ratio of rostral segments 1:2.10:0.52 in both sexes. Neck dark with 1+1 reddish lateral spots.

Legs slender; fore femora slightly incrassate, about three times as long as wide at mid-length, ventrally with a row of small, blunt denticles more evident in the distal half; mid and hind femora comparatively more cylindrical, with finer denticulation or smooth. Tibiae straight, lacking strong armature; apices with two well-developed lateral spines. In males, small spongy fossulae present on the ventral apices of fore and mid tibiae; hind tibiae without fossulae. In females, spongy fossulae absent or extremely reduced on all tibiae.

Pronotum with fore lobe slightly granulose; discal tubercles indistinct; lateral tubercles absent. Hind lobe rugose, with humeral angles rounded and slightly elevated. General color dark brown to black, with lateral margins orange-red, the pale area extending onto the humeral disc and the collar, including the anterolateral angles. Overall aspect of the pronotum marked by a dark disc contrasting with the light anterior and lateral borders, as well as the lateral portions of the hind margin, which are bordered by a reddish line of variable width. Scutellum rugose, bearing delicate irregular carinae that delimit the central depression; apical process subcylindrical, slightly shorter than the main body of the scutellum, with a rounded, declinate apex.

Hemelytra not reaching the abdominal apex. Corium with sparse, short and inconspicuous setae; basal triangle reddish-orange, followed by a broad central dark-brown area spanning the full width of the corium; a smaller subapical orange-red spot is present, with the extreme apex dark. Clavus dark on the basal half, becoming light brown apically, sometimes with orange spots visible near the base. General color of abdomen uniformly dark; ventrally with pilosity and delicately striate transversally. Spiracles close to but not contiguous with connexival suture. Connexival segments dorsally and ventrally dark, with pale markings on the posterior half or third. The pale areas of the connexivum consistently extend ventrally to the level of the spiracle across all segments, such that a line projected laterally from the spiracle intersects the pale portion of the connexivum.

### Distribution

## Discussion

In this study, we integrated morphometric and molecular evidence to reassess taxonomic boundaries within *Triatoma sanguisuga* sensu lato and to test whether Florida populations currently referred to as *T. sanguisuga* correspond to *Triatoma ambigua* Neiva, 1911 (stat. rev.). The name was originally introduced as *T. sanguisuga* var*. ambigua* from Florida material (Neiva, 1911) ^24^. Under the International Code of Zoological Nomenclature (ICZN), names proposed at infrasubspecific rank require careful evaluation of availability and species-group status; we therefore revisit the nomenclatural history and provide an updated diagnosis and redescription based on integrative evidence. Because species and subspecies are both species-group taxa under the ICZN, changes in rank do not alter authorship of the species-group name.

Our results show that specimens from Florida comprise a strongly divergent mitochondrial lineage (GMYC Group 1), with K2P distances >7.5% from all other sampled groups. This pattern is concordant with ITS-2 differentiation and with consistent morphometric separation. Linear morphometrics, LDA, and random-forest analyses recovered stable differences in overall size and cephalic traits between populations sampled in Florida vs other states, particularly synthlipsis width, and connexival coloration provided an additional qualitative diagnostic character. Taken together, these congruent datasets support revalidation of *T. ambigua* Neiva, 1911 (stat. rev.) as a distinct species within the *T. sanguisuga* complex ^24^.

Notably, several sequences from Texas deposited in GenBank as *T. indictiva* or *Triatoma sp*. clustered within Group 3, which is composed predominantly of *T. sanguisuga* specimens. These localities fall within the known zone of sympatry between *T. sanguisuga* and *T. indictiva* in south-central Texas (8,28), raising the possibility of misidentification, hybridization, or incomplete lineage sorting. Further integrative taxonomic work focusing on Texas populations is needed to resolve species boundaries in this region.

Our findings are consistent with previous reports of substantial mitochondrial diversity and population structuring within *T. sanguisuga*. Along the Georgia barrier islands, ^28^ found non-overlapping COII haplotypes and evidence of limited inter-island gene flow. De la Rúa et al. (2011) reported deeply divergent *cytb* and 16S haplotypes within a single Louisiana locality ^29^, with divergence magnitudes comparable to those reported between some recognized triatomine species; subsequent work integrating nuclear ITS-2 and morphometrics further indicated complex intraspecific structure ^30^. Comparable patterns of cryptic diversity have been documented in other triatomine groups, including the *Triatoma brasiliensis* complex and related North/Central American taxa, where combined molecular and morphometric evidence has helped refine species boundaries ^31,33,34^. By linking one divergent lineage to Neiva’s *ambigua* form and providing an updated diagnosis based on concordant genetic and morphological evidence, our study extends this integrative framework to the *T. sanguisuga* complex.

Ecologically, available data suggest that the *T. ambigua* lineage operates primarily at the human-wildlife interface, with recurrent intrusion into domestic and peridomestic environments. Current ecological observations derive primarily from Florida, where specimens assigned here to *T. ambigua* were collected in and around dwellings across 23 counties, with substantial *T. cruzi* infection (~29.5%) and broad host use including humans, domestic animals, and synanthropic wildlife ^8,11,35^. This pattern differs from reports from some *T. sanguisuga* population, including Texas kennel/peridomestic environments where triatomines can occur at high abundance in man-made structures ^6,36^. The combination of genetic distinctiveness, consistent morphological differentiation, and repeated house intrusion without clear evidence of stable domiciliation is consistent with intrusive triatomine ecologies described in Latin America ^20–22^.

Differences in sylvatic versus peridomestic ecology have important implications for transmission. In Florida, *T. ambigua* appears to link sylvatic reservoirs (notably raccoons and opossums) with domestic spaces, with triatomines and infected mammals both documented in peridomestic settings ^8,11,35^. In Texas and Louisiana, *T. sanguisuga* and related lineages are also associated with peridomestic environments and, in some settings, can show high bug abundance and substantial *T. cruzi* infection prevalence ^6,15,16,36^. These contrasts suggest that lineages within the *T. sanguisuga* complex may differ in propensity for domiciliary intrusion, host-use patterns, and transmission pathways linking sylvatic and domestic cycles. Although we did not directly measure defecation timing or metacyclogenesis, both traits vary among triatomine species and are relevant to vectorial capacity; comparative work on *T. ambigua* versus other species in the *T. sanguisuga* complex should prioritize these traits.

Our genetic results may help explain the pronounced geographic heterogeneity in *T. cruzi* infection prevalence across the *T. sanguisuga* complex. *T. ambigua* showed intermediate infection prevalence (~29.5%), whereas *T. sanguisuga* from some Texas peridomestic/kennel settings has shown substantially higher prevalence in specific surveys ^6,8,36^. Louisiana populations, represented mainly by our GMYC Groups 2 and 4, have also shown high infection prevalence and marked local mitochondrial structure ^15,16,29^. In our dataset, the four *cytb* groups broadly align with regional foci (Florida, Louisiana/central states, Texas, and mixed Texas-Louisiana lineages), suggesting that transmission systems are geographically and evolutionarily structured. Integrating vector lineage assignments with parasite DTU genotyping and blood-meal profiling will be important to test whether vector genetics, host communities, and parasite population structure jointly drive geographic differences in infection prevalence and exposure risk.

The distinctiveness of *T. ambigua* may reflect deeper biogeographic history. During the Pleistocene, recurrent sea-level fluctuations fragmented the current-day Florida peninsula into isolated upland refugia, a process associated with divergence and endemism in multiple taxa ^37,38^. Although we did not conduct formal phylogeographic modeling, the combination of deep genetic divergence, occurrence predominantly in Florida in our sampling (with one morphologically consistent specimen from Georgia), and consistent morphological differentiation is compatible with historical isolation of Florida populations followed by divergence from more northerly and western *T. sanguisuga* lineages. Expanded geographic sampling, especially from southern Alabama, Georgia, South Carolina, and other southeastern states adjacent to Florida, together with genome-scale datasets, will be necessary to test this hypothesis and to assess whether additional cryptic lineages occur within the *T. sanguisuga* complex as defined here.

Our integrative framework aligns with current triatomine systematics, in which species hypotheses are evaluated using concordant molecular and morphological evidence, especially in groups with overlapping characters and complex evolutionary histories ^30,31,33,34,39^. Nevertheless, several limitations remain. First, most genetic inference here relies on mtDNA plus ITS-2; although marker concordance and morphology support *T. ambigua*, genome-scale population analyses will be needed to test lineage boundaries and ongoing gene flow more rigorously ^14,29^. Second, geographic coverage is still incomplete, particularly in states adjacent to Florida (e.g., southern Alabama, Georgia) and across the broader southeastern U.S. range, so additional diversity may remain unsampled. Third, although preserved triatomine specimens are widely used in morphometric studies, measurement error can still arise from specimen preparation, orientation, photography, and landmark placement, particularly for head-based traits; we reduced this risk through standardized preparation and emphasis on relative traits, but comparison with newly collected material would further strengthen inference ^40–43^. Finally, we did not directly test feeding-defecation behavior or experimental transmission competence, traits known to vary across triatomines and to influence vectorial capacity, and these should be prioritized in comparative work on *T. ambigua* and other *T. sanguisuga* lineages ^44–46^.

Despite these limitations, recognizing *T. ambigua* as a species distinct from *T. sanguisuga* has practical implications for Chagas disease research and surveillance in North America. Taxonomically, it stabilizes the name proposed by Neiva (1911) and provides clear diagnostic characters connexivum pattern, synthlipsis width thresholds, that can be applied in field and museum settings; overall body size tends to be smaller in *T. ambigua*, but this character should be used with caution given individual variation. Epidemiologically, it emphasizes that what has historically been treated as a single widespread vector comprises at least two species, with differing geographic ranges, ecological associations and infection profiles. This recognition can improve mapping of *T. cruzi* risk, help interpret differences in exposure and disease burden between states such as Florida, Texas and Louisiana, and guide ecologically tailored vector-control strategies ^24^.

### Geographic Sampling Limitations.

Our molecular and morphometric analyses relied primarily on specimens collected from Florida, with limited representation from adjacent states in the southeastern United States (e.g., one specimen from Georgia). The apparent geographic clustering of *T. ambigua* within Florida may reflect sampling bias rather than true distributional boundaries. Future studies incorporating specimens from southern Alabama, Georgia, and other Gulf Coast states are needed to determine whether *T. ambigua* extends beyond the areas sampled in this study. Political boundaries do not constrain species distributions, and we caution against interpreting our results as evidence that *T. ambigua* is restricted to Florida.

More broadly, our work underscores the value of community science and collaborative field program in revealing cryptic diversity within vector complexes. Specimens from Texas and Florida citizen-science initiatives, combined with targeted collections, were critical for detecting the unusual genetic and morphological profile of *T. ambigua* and for motivating a formal taxonomic re-evaluation ^7,8,47^. Future research should extend this integrative framework across the full range of the *T. sanguisuga* complex as define here, with particular emphasis on under sampled areas adjacent to Florida (southern Alabama, Georgia, South Carolina) where the distributional boundaries of *T. ambigua* and *T. sanguisuga* sensu stricto remain undefined and where potential contact or hybrid zones may exist, such study should explicitly compare ecology, behavior, *T. cruzi* DTUs, host utilization and human exposure among lineages. Such work will not only refine our understanding of the evolution of North American triatomines but also improve the evidence base for preventing Chagas disease transmission in the region.

## Material and Methods

### Sample Collection and Origin

For the molecular analysis a total of 58 adult specimens of *Triatoma sanguisuga* were analyzed, identified based on the protocol described by Lent and Wygodzinsky ^23^. Specimens were collected from various southeastern U.S. states, primarily Texas and Florida. In total, samples are representative of 54 U.S. counties of 7 states. These triatomine specimens were voluntarily submitted to community science programs at Texas A&M University ^47^ and the University of Florida ^8^.

**Figure 12.**
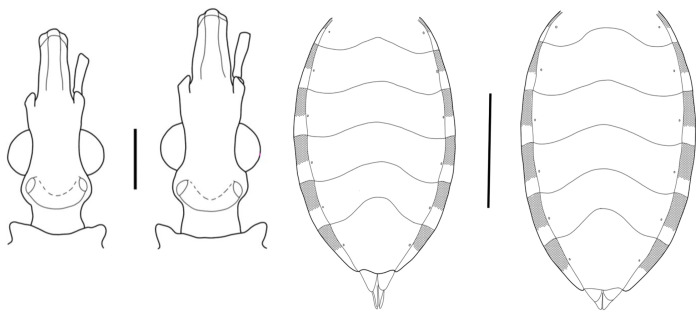
Diagnostic morphological comparison between *Triatoma ambigua* stat. nov. and *T. sanguisuga*. (A) Dorsal view of the head showing differences in synthlipsis width; *T. ambigua* (left) and *T. sanguisuga* (right). Scale bar = 1 mm. (B) Ventral view of the abdomen showing connexivum coloration pattern; *T. ambigua* stat. nov. (left) and *T. sanguisuga* (right). Scale bar = 5 mm.

For morphometric analysis a total of 82 adult specimens provided by field collections in Florida and Texas as well submissions from community science programs as described, in addition to submitted curated specimens to the Florida Department of Agricultural and Consumer Services, Florida State Collection of Arthropods (FSCA) located in Gainesville, Florida, USA, were utilized for this analysis. A total of 49 adult specimens (n=29 female; n=20 male) were included in the assessment from across 17 counties in Florida. Field collected specimens from our team took place from 2023 to 2024. Archived specimens housed at the FSCA collection were collected from as early as 1905 until 2006. The 33 adult specimens from outside Florida (n=21 female; n=12 male) were obtained from across 25 counties/parishes in an additional 13 other states (Figure 12). These specimens from outside Florida were from field collections in Texas and Missouri from 2018 until 2024 and included submitted specimens to FSCA collection from as early as 1946 until 2024.

### DNA extraction, PCR Amplification, Phylogenetic and Genetic Diversity Analyses

Genomic DNA was extracted from leg tissues using the DNeasy Blood & Tissue Kit (QIAGEN) following the manufacturer’s instructions with minor modifications. Extracted DNA was stored at −20°C for downstream applications. DNA concentration was measured with a NanoDrop 2000 spectrophotometer (Thermo Fisher Scientific). DNA integrity was assessed via electrophoresis on 0.8% agarose gels stained with GelRed^®^. The mitochondrial *cytb* gene was amplified using primers CYTB7432F and CYTB7433R ^48^. PCR reactions were conducted in a thermocycler using the following program: initial denaturation at 95°C for 5 minutes, 35 cycles of 95°C for 30 seconds, 47°C for 30 seconds, and 72°C for 60 seconds, followed by a final extension at 72°C for 10 minutes. For the nuclear ITS-2 marker, a new pair of primers were designed, due to the inability to use primers from Marcilla et al (2021). Forward (5’-ATTCTATCCAAGGGCCACGC −3’) and Reverse (5’- TCCCCTTTCGGATATTCAGGTC −3’) primers were designed based on the 5.8S rDNA and 28S rDNA sequence from several Triatominae species respectively^34^. The PCR program was as follows: initial denaturation at 95°C for 5 minutes, 35 cycles of 95°C for 30 seconds, 62°C for 30 seconds, and 72°C for 70 seconds, followed by a final extension at 72°C for 10 minutes and both validated by electrophoresis on 0.8% agarose gels stained with GelRed^®^, and were sequenced using Sanger technology at Macrogen Inc. (South Korea). Resulting sequences were manually inspected using FinchTV v1.4.0 ^49^.

Curated *cytb* sequences were used to perform a blastn search on GenBank ^50^. As a result 96 publicly available *cytb* sequences were retrieved, including 64 identified as *T. sanguisuga*, eight as *T. indictiva*, and 24 as *Triatoma* sp. These *T. indictiva* and *Triatoma* sp. were included in the following analyses due to the high identity with our sequences. A complete list of all cytb and ITS-2 sequences and their accession numbers can be found in Supplementary Table 3. All the dataset was aligned with MAFFT v.7.453^51^ and haplotype identification was carried out using the *ape* v.5.7-1 ^52^ and *pegas* v.1.2 ^53^ R packages. Species delimitation was performed with four approaches: Hierarchical Bayesian Analysis of Population Structure (HierBAPS) implemented in the *rhierBAPS* v.1.1.3 ^54^ R package, ABGD implemented in the command line version ^55^, mPTP implemented in the web server (https://mptp.h-its.org/#/tree) with the optimized Maximum likelihood tree as input ^56^ and Generalized Mixed Yule-Coalescent (GMYC) ^57^ analysis using *splits* v1.0 ^58^. The GMYC analysis required an ultrametric tree, which was generated in BEAST v1.10 ^59^ using the TN93+G+I substitution model (selected based on BIC using the model finder option in IQtree, see later), local clock model, and Bayesian Skygrid tree prior. Markov Chain Monte Carlo (MCMC) simulations were run for 400 million generations, and convergence was assessed with Tracer v1.7 ^60^.

In addition to the Bayesian inference (BI) tree using BEAST, phylogenetic relationships were inferred using two more methodologies: Maximum Likelihood (ML) with IQ-TREE v1.6.12 ^61^, and maximum parsimony (MP) with *phangorn* v.2.11.1 ^62^ package implemented in R. For ML, the best-fit substitution model was selected using Bayesian Information Criterion (BIC), and node support was assessed with 1000 bootstrap pseudoreplicates. For MP, support was calculated also with 1000 bootstrap pseudoreplicates. Population genetic diversity indices, including nucleotide diversity (π), haplotype diversity (Hd), and segregation sites (S), were calculated for the total dataset and for each group identified by GMYC. Neutrality tests (Tajima’s D and Ramos-Onsins and Rozas’ R2) were performed to assess population expansion. Genetic distances were calculated under the Kimura-2-parameter model, and intergroup distances were compared. Distances between haplotypes were represented as a heatmap using the R packages *ggtree* ^63^ and *ggplot2* ^64^. To visualize haplotype relationships, a Minimum Spanning Tree (MST) haplotype network was constructed using PopART ^65^.

For those individuals with both markers (n=23), we concatenate sequences of *cytb* and ITS-2 to obtain a BI tree using each gene as a partition using BEAST v1.10 ^59^. For *cytb* gene fragment, the TN93 substitution model was selected as the best-fitted, while for ITS-2 marker, the HKY substitution model was the chosen one. A local clock model was established for both sets of markers, along with a Coalescent tree prior assuming a constant population size, chosen as the most suitable approach for the size of the dataset. To employ the same individual for both markers, a *Triatoma dimidiata* specimen from Veracruz, Mexico was used as an outgroup (NCBI BioSample SAMN60274295). Both markers were obtained mapping the NGS reads to the *T. sanguisuga* markers using Novoplasty (available at https://github.com/ndierckx/NOVOPlasty).

Phylogenetic trees and genetic distance heatmaps were edited and visualized using *ggtree* v3.8.2 ^63^ and *ggplot2* v3.5.1 ^64^ in R. Geographic distributions of samples were mapped to highlight spatial genetic structuring using R packages *ggplot2* v3.5.1, *dplyr* v1.1.4 ^66^, *tidyr* v1.3.1 ^67^, *maps* v3.4.0 ^68^, *scatterpie* v0.2.1 ^69^.

### Morphometric analysis

Following genetic analyses, which identified a distinct group (Group 1) composed exclusively of specimens from Florida showing substantial genetic divergence and the highest haplotype diversity, a targeted morphological examination was carried out to determine whether this genetic differentiation was reflected in consistent phenotypic traits. To identify morphometric differences that supported the differentiation of Florida samples, eighteen morphological characters were investigated (in alphabetical order; abbreviations in parentheses): 1st antennomere (ANT1), 1st labial article (LAB1), 2nd antennomere (ANT2), 2nd labial article (LAB2), 3rd antennomere (ANT3), 3rd labial article (LAB3), 4th antennomere (ANT4), ante-ocular region (AOR), head length (HL), length of pronotum (PL), length of scutellum (SL), post-ocular region (POR), synthlipsis (SYN), total length (TL), width across eyes (WE), width of abdomen (WA), width of eye (EW), and width of pronotum (PW). This morphometric analysis assessment modeled other such triatomine new species delineation as described by Lima-Cordón et al (2019) ^33^. We assessed 49 previously identified *T. sanguisuga* from Florida specimens collected in the field from 2023 to 2024 and archived specimens housed at the Florida Department of Agricultural and Consumer Services, Florida States Collection of Arthropods (FSCA) from as early as 1905 until 2006. Specimens from outside Florida (n=33) were also assessed from field collections in Texas and Missouri from 2018 until 2024 and submitted specimens to FSCA from as early as 1946 until 2024. All morphometric data collected from 82 specimens used in this analysis are found in Supplementary Table 4. Each morphometric character was measured in millimeters and recorded to the one-hundredth decimal of a millimeter. A Leica encoded stereo digital microscope (Leica microsystems; Model M205C) with 20-megapixel color complementary metal-oxide semiconductor camera (Leica miscrosystems; Model DMC5400) was used in conjunction with the Leica application suite X software platform to capture each image and measurement.

To assess overall morphological clustering, we first evaluated covariance homogeneity across all 18 characters using Box’s M test from the biotools package 70. We then applied a backward-elimination procedure, at each iteration removing the single character whose exclusion maximized the Box’s M p-value, until the test was no longer significant (p > 0.05). This process excluded ANT1, ANT3, ANT4, LAB1, LAB2, LAB3, and EW. The retained subset of characters satisfying the homogeneity assumption was subjected to linear discriminant analysis (LDA) using the MASS package in Program R ^71^, with state specified as the grouping factor. LDA coefficients were examined to quantify each trait’s relative contribution to group separation. Given that male triatomines are generally smaller than females ^23^, LDA analyses were performed separately by sex. A series of non-parametric statistical analyses separately for males and females. All morphological characters were analyzed using the unpaired Wilcoxen-test to assess whether median trait values differed significantly among Florida and non-Florida samples within each sex. Characters with adjusted p-values below 0.05 were considered significantly different.

To identify diagnostic traits for the Florida population, we prioritized morphological characters that (i) exhibited statistically significant differences among morphotypes and (ii) had high absolute coefficients in linear discriminant analysis (LDA), indicating strong discriminatory power. Receiver Operating Characteristic (ROC) curve analysis was then used to assess the diagnostic utility of selected traits. Optimal threshold values were determined using Youden’s Index, which identifies the point that maximizes the combined sensitivity and specificity. This combined approach guided the selection of candidate traits supporting morphological species delimitation.

## Supplementary Material

This is a list of supplementary files associated with this preprint. Click to download.

• Supplementarytable1.xlsx

• Supplementarytable2.xlsx

• Supplementarytable3.xlsx

• Supplementarytable4.xlsx

## Figures and Tables

**Figure 6.**
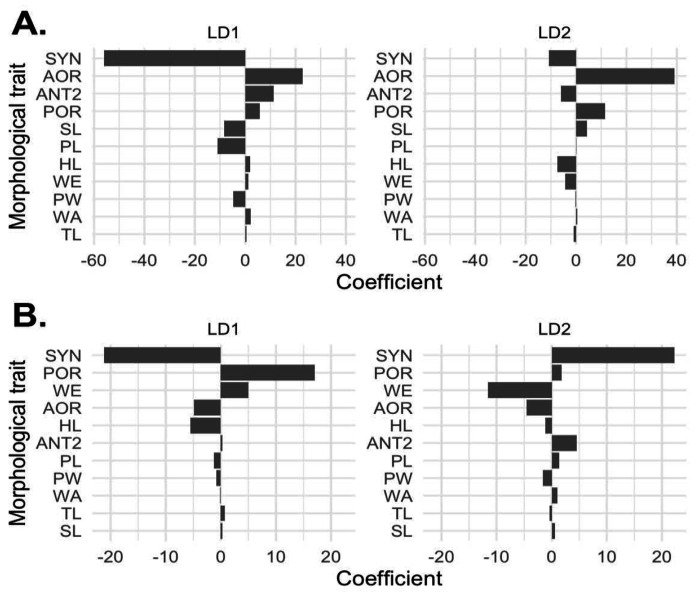
Linear Discriminant Analysis (LDA). Loading coefficients for morphological traits in male (top) and female (bottom) specimens. Traits are ordered by the absolute value of their contribution to each discriminant function, with separate panels for each LD1 and LD2. Positive and negative coefficients indicate the direction and magnitude of each trait’s influence on group separation. Differences in loading patterns between sexes highlight sex-specific morphological variation driving the discrimination among states.

**Figure 8.**
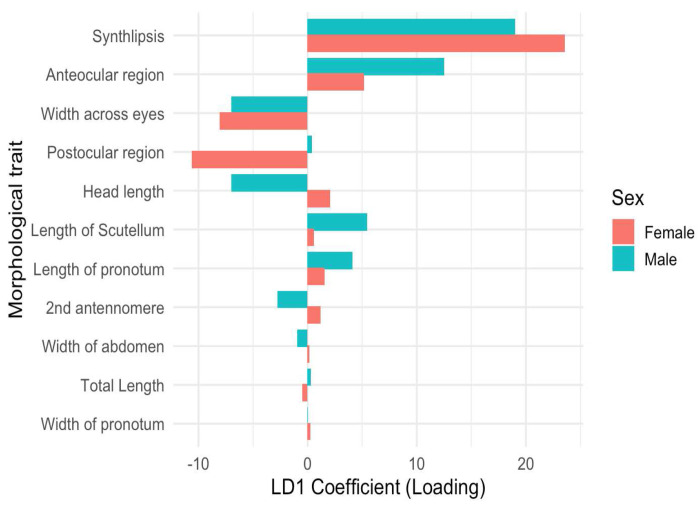
Linear discriminant analysis (LDA). Loadings for morphological traits contributing to species separation along the primary axis (LD1) in males and females. Traits are ordered by the absolute value of their LD1 coefficients, illustrating the relative importance of each morphological character in discriminating between *T. ambigua* and *T. sanguisuga*. Bars represent the magnitude and direction of the loadings, with colors indicating sex.

**Figure 9.**
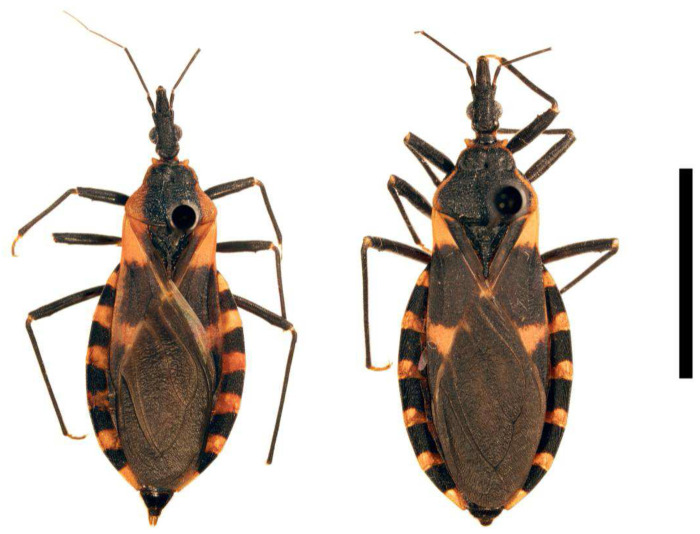
Dorsal view of *T. ambigua* (left) and *T. sanguisuga* (right). Scale bar = 1 cm.

**Figure 10.**
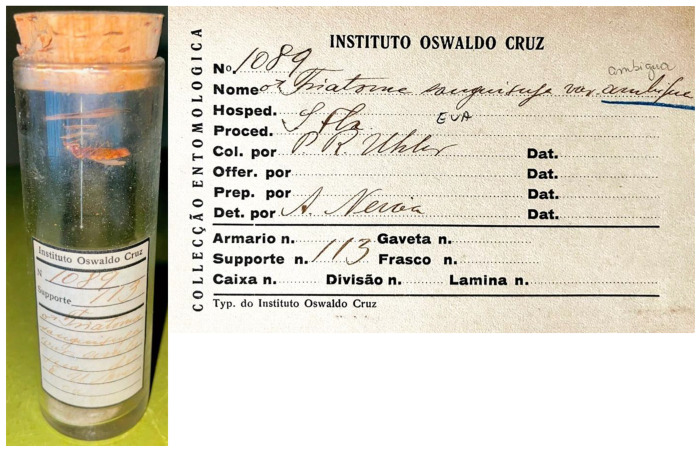
Lectotype of *Triatoma ambigua* (Neiva, 1911) (CEIOC 10020; CCP1089). (A) Lectotype specimen preserved in sealed glass tube (Instituto Oswaldo Cruz). (B) Associated original label showing register number “1089”, identification as “*T. sanguisuga* var. *ambigua*”, collector P.R. Uhler, and determination by A. Neiva.

**Table 1.** Mitochondrial *cytb* gene fragment sequence diversity in *Triatoma sanguisuga* groups.

	N	S	Nh	Hd	*π*	D	D P-val	R2	R2 P-val
Group 1	19	37	15	0.964	0.016	−1.184	0.236	0.084	0.036*
Group 2	60	69	43	0.980	0.023	−0.960	0.336	0.072	0.199
Group 3	66	49	37	0.896	0.012	−1.509	0.131	0.052	0.073
Group 4	28	33	18	0.915	0.016	−0.483	0.628	0.096	0.253

Total	173	116	113	0.980	0.046	0.121	0.903	0.089	0.608

N: number of specimens analyzed. S: number of segregating sites. Nh: number of haplotypes. Hd: haplotype diversity. *π*: nucleotide diversity. D: Tajima’s D neutrality test value. D P-val: p-value fro Tajima’s D neutrality test. R2: Ramos–Onsins–Rozas neutrality test value. R2 P-val: p-value from R2 neutrality test.

**Table 2.** Mitochondrial *cytb* gene fragment genetic pairwise distance (k2-p corrected) between GMYC groups. values are depicted in percentage.

	Group 1	Group 2	Group 4	Group 3
Group 1	1.73	8.65	8.7	7.65
Group 2	8.65	2.72	5.94	6.54
Group 4	8.7	5.94	1.7	4.5
Group 3	7.65	6.54	4.5	1.29

**Table 3.** Morphometric measurements from 18 anatomical characters specimens from Florida and states.

	Florida		Non-Florida	
	♂ (mm)	♀ (mm)	♂ (mm)	♀ (mm)
Total Length^†^	18.16 (1.16)	19.65 (1.50)	20.78 (0.97)	22.30(1.16)
Width of abdomen^†^	6.39(0.77)	7.22 (0.64)	7.39 (0.60)	7.88 (0.64)
Head length^†^	2.97 (0.19)	3.09 (0.21)	3.31 (0.20)	3.48 (0.14)
Width across eyes*	1.68 (0.11)	1.68 (0.10)	1.76 (0.12)	1.86 (0.09)
Anteocular region^†^	1.61 (0.08)	1.67 (0.11)	1.84 (0.11)	1.95 (0.10)
Postocular region	0.62 (0.07)	0.63 (0.08)	0.65 (0.09)	0.69 (0.07)
Width of eye	0.46 (0.04)	0.44 (0.04)	0.45 (0.03)	0.46 (0.04)
Synthlipsis^†^(intraocular distance	0.76 (0.05)	0.79 (0.05)	0.86 (0.07)	0.93 (0.04)
Width of pronotum^†^	4.28 (0.37)	4.46 (0.32)	4.96 (0.38)	5.21 (0.38)
Length of pronotum^†^	3.00 (0.22)	3.13 (0.24)	3.54 (0.20)	3.68 (0.24)
Length of Scutellum^†^	2.15 (0.18)	2.30 (0.22)	2.49 (0.17)	2.58 (0.22)
1st antennomere^†^	0.66 (0.07)	0.66 (0.07)	0.80 (0.08)	0.81 (0.10)
2nd antennomere*	2.45 (0.22)	2.28 (0.30)	2.71 (0.22)	2.65 (0.29)
3rd antennomere^‡^	1.68 (0.15)	1.64 (0.26)	1.98 (0.14)	1.91 (0.25)
4th antennomere	1.50 (0.22)	1.33 (0.44)	1.30 (0.69)	1.22 (0.59)
1st labial article^†^	1.16 (0.11)	1.17 (0.11)	1.36 (0.09)	1.34 (0.12)
2nd labial article^†^	2.25 (0.09)	2.27 (0.18)	2.46 (0.09)	2.59 (0.11)
3rd labial article*	0.56 (0.05)	0.56 (0.05)	0.61 (0.08)	0.65 (0.04)
**n**	20	29	12	21

† Significant difference between Florida and non-Florida *T. sanguisuga* in both sexes (*p* < 0.05, unpaired *t*-test).

* Significant difference in females only (*p* < 0.05).

‡ Significant difference in males only (*p* < 0.05). Values are mean with standard deviation shown in parenthesis.

## Data Availability

The datasets generated and/or analysed during the current study are available in Supplementary Table 1, Supplementary Table 2, Supplementary Table 3, and Supplementary Table 4. All genomic sequences generated in this report are found in Supplementary Table 3 with corresponding accession numbers and voucher identification which were submitted to NIH GenBank (https://www.ncbi.nlm.nih.gov/nuccore/ ), under numbers PZ124889-PZ124937 for cytb; PZ134240-PZ134262 for ITS-2; BioSample SAMN60274295.

## References

[1] Beatty N. L., Hamer G. L., Moreno-Peniche B., Mayes B. & Hamer S. A. Chagas Disease, an Endemic Disease in the United States. Emerg. Infect. Dis. 31, 1691–1697 (2025). 40866797 10.3201/eid3109.241700 PMC12407112

[2] Lidani K. C. F. Chagas Disease: From Discovery to a Worldwide Health Problem. Front. Public Health 7, 166 (2019). 31312626 10.3389/fpubh.2019.00166 PMC6614205

[3] Bern C., Messenger L. A., Whitman J. D. & Maguire J. H. Chagas Disease in the United States: a Public Health Approach. Clin. Microbiol. Rev. 33, e00023–19 (2019). 31776135 10.1128/CMR.00023-19 PMC6927308

[4] Agudelo Higuita N. I. Chagas disease in the United States: a call for increased investment and collaborative research. Lancet Reg. Health - Am. 34, 100768 (2024). 38798947 10.1016/j.lana.2024.100768 PMC11127192

[5] Beatty N. L. & Klotz S. A. Autochthonous Chagas Disease in the United States: How Are People Getting Infected? Am. J. Trop. Med. Hyg. 103, 967–969 (2020). 32602437 10.4269/ajtmh.19-0733 PMC7470559

[6] Curtis-Robles R., Hamer S. A., Lane S., Levy M. Z. & Hamer G. L. Bionomics and Spatial Distribution of Triatomine Vectors of Trypanosoma cruzi in Texas and Other Southern States, USA. Am. J. Trop. Med. Hyg. 98, 113–121 (2018). 29141765 10.4269/ajtmh.17-0526 PMC5928729

[7] Beatty N. L., Forsyth C. J., Burkett-Cadena N. & Wisely S. M. Our Current Understanding of Chagas Disease and Trypanosoma cruzi Infection in the State of Florida — an Update on Research in this Region of the USA. Curr. Trop. Med. Rep. 9, 150–159 (2022).

[8] Beatty N. L. Field evidence of Trypanosoma cruzi infection, diverse host use and invasion of human dwellings by the Chagas disease vector in Florida, USA. PLoS Negl. Trop. Dis. 19, e0012920 (2025). 40623053 10.1371/journal.pntd.0012920 PMC12233274

[9] Beatty N. L. Integrated pest management strategies targeting the Florida kissing bug, Triatoma sanguisuga: Preventing this vector of Chagas disease from invading your home. Curr. Res. Parasitol. Vector-Borne Dis. 4, 100144 (2023). 37841307 10.1016/j.crpvbd.2023.100144 PMC10570570

[10] Torhorst C. W. Trypanosoma cruzi infection in mammals in Florida: New insight into the transmission of T. cruzi in the southeastern United States. Int. J. Parasitol. Parasites Wildl. 21, 237–245 (2023). 37575667 10.1016/j.ijppaw.2023.06.009 PMC10422094

[11] Torhorst C. W., White Z. S., Bhosale C. R., Beatty N. L. & Wisely S. M. Identification of the parasite, Trypanosoma cruzi, in multiple tissues of epidemiological significance in the Virginia opossum (Didelphis virginiana): Implications for environmental and vertical transmission routes. PLoS Negl. Trop. Dis. 16, e0010974 (2022). 36534706 10.1371/journal.pntd.0010974 PMC9810149

[12] CDC. About the National Center for Emerging and Zoonotic Infectious Diseases. National Center for Emerging and Zoonotic Infectious Diseases (NCEZID) https://www.cdc.gov/ncezid/about/index.html (2025).

[13] Peterson J. K. First Report of Chagas Disease Vector Species Triatoma sanguisuga (Hemiptera: Reduviidae) Infected with Trypanosoma cruzi in Delaware. Am. J. Trop. Med. Hyg. 110, 925–929 (2024). 38531096 10.4269/ajtmh.23-0915 PMC11066352

[14] Peterson J. K., MacDonald M. L. & Ellis V. A. First whole-genome sequence of *Triatoma sanguisuga* (Le Conte, 1855), vector of Chagas disease. G3 Genes Genomes Genet. 15, jkae308 (2025). 10.1093/g3journal/jkae308 PMC11917478 39739582

[15] Cesa K., Caillouët K. A., Dorn P. L. & Wesson D. M. High *Trypanosoma cruzi* (Kinetoplastida: Trypanosomatidae) Prevalence in *Triatoma sanguisuga* (Hemiptera: Redviidae) in Southeastern Louisiana. J. Med. Entomol. 48, 1091–1094 (2011). 21936329 10.1603/me10195 PMC3544525

[16] Moudy R. M. Factors associated with peridomestic Triatoma sanguisuga (Hemiptera: Reduviidae) presence in southeastern Louisiana. J. Med. Entomol. 51, 1043–1050 (2014). 25276935 10.1603/me13234

[17] Curtis-Robles R., Auckland L. D., Snowden K. F., Hamer G. L. & Hamer S. A. Analysis of over 1500 triatomine vectors from across the US, predominantly Texas, for Trypanosoma cruzi infection and discrete typing units. Infect. Genet. Evol. 58, 171–180 (2018). 29269323 10.1016/j.meegid.2017.12.016

[18] Balasubramanian S. Characterization of triatomine bloodmeal sources using direct Sanger sequencing and amplicon deep sequencing methods. Sci. Rep. 12, 10234 (2022). 35715521 10.1038/s41598-022-14208-8 PMC9205944

[19] Dumonteil E., Tu W., Jiménez F. A. & Herrera C. Ecological interactions of *Triatoma sanguisuga* (Hemiptera: Reduviidae) and risk for human infection with *Trypanosoma cruzi* (Kinetoplastida: Trypanosomatidae) in Illinois and Louisiana. J. Med. Entomol. 61, 1282–1289 (2024). 38373261 10.1093/jme/tjae017

[20] Dumonteil E. Interactions among *Triatoma sanguisuga* blood feeding sources, gut microbiota and *Trypanosoma cruzi* diversity in southern Louisiana. Mol. Ecol. 29, 3747–3761 (2020). 32749727 10.1111/mec.15582

[21] Dumonteil E. Detailed ecological associations of triatomines revealed by metabarcoding and next-generation sequencing: implications for triatomine behavior and Trypanosoma cruzi transmission cycles. Sci. Rep. 8, 4140 (2018). 29515202 10.1038/s41598-018-22455-x PMC5841364

[22] Waleckx E., Suarez J., Richards B. & Dorn P. L. *Triatoma sanguisuga* Blood Meals and Potential for Chagas Disease, Louisiana, USA. Emerg. Infect. Dis. 20, 2141–2143 (2014). 25418456 10.3201/eid2012.131576 PMC4257814

[23] Lent H. & Wygodzinsky P. W. Revision of the Triatominae (Hemiptera, Reduviidae), and their significance as vectors of Chagas’ disease. Bull. Am. Mus. Nat. Hist. 163, 123–520 (1979).

[24] Neiva A. Notas de entomolgia medica e descripcao de duas novas especies de Triatomas norte-americans. Brasil-Médico. 421–423 (1991).

[25] Mead FW. The Blood-sucking Conenose. Ent Cir. 33, (1965).

[26] Usinger R. L. The Triatominae of North and Central America and the West Indies and their Public Health Significance. in (1944).

[27] Kjos S. A. Identification of Bloodmeal Sources and *Trypanosoma cruzi* Infection in Triatomine Bugs (Hemiptera: Reduviidae) From Residential Settings in Texas, the United States. J. Med. Entomol. 50, 1126–1139 (2013). 24180119 10.1603/me12242 PMC3932564

[28] Roden A. E., Champagne D. E. & Forschler B. T. Biogeography of Triatoma sanguisuga (Hemiptera: Reduviidae) on Two Barrier Islands off the Coast of Georgia, United States. J. Med. Entomol. 48, 806–812 (2011). 21845939 10.1603/me11049

[29] De La Rua N., Stevens L. & Dorn P. L. High genetic diversity in a single population of Triatoma sanguisuga (LeConte, 1855) inferred from two mitochondrial markers: Cytochrome b and 16S ribosomal DNA. Infect. Genet. Evol. 11, 671–677 (2011). 21333758 10.1016/j.meegid.2011.02.009

[30] De La Rúa N. M. Towards a phylogenetic approach to the composition of species complexes in the North and Central American Triatoma, vectors of Chagas disease. Infect. Genet. Evol. 24, 157–166 (2014). 24681261 10.1016/j.meegid.2014.03.019 PMC4096843

[31] Kieran T. J. Ultraconserved elements reconstruct the evolution of Chagas disease-vectoring kissing bugs (Reduviidae: Triatominae). Syst. Entomol. 46, 725–740 (2021).

[32] Rodrigues J. M. D. S., Moreira F. F. F., Deckert J. & Galvão C. List of the type specimens of Triatominae (Hemiptera: Heteroptera: Reduviidae) in the Hemimetabola Collection of the Museum für Naturkunde, Berlin. Zootaxa 4809, (2020). 10.11646/zootaxa.4809.2.5 33055938

[33] Lima-Cordón R. A. Description of Triatomahuehuetenanguensis sp. n., a potential Chagas disease vector (Hemiptera, Reduviidae, Triatominae). ZooKeys 51–70 (2019) doi:10.3897/zookeys.820.27258. PMC6361876 30728739

[34] Marcilla A. The ITS-2 of the nuclear rDNA as a molecular marker for populations, species, and phylogenetic relationships in Triatominae (Hemiptera: Reduviidae), vectors of Chagas disease. Mol. Phylogenet. Evol. 18, 136–142 (2001). 11161750 10.1006/mpev.2000.0864

[35] Torhorst C. W. Trypanosoma cruzi infection in mammals in Florida: New insight into the transmission of T. cruzi in the southeastern United States. Int. J. Parasitol. Parasites Wildl. 21, 237–245 (2023). 37575667 10.1016/j.ijppaw.2023.06.009 PMC10422094

[36] Busselman R. E. Abundant triatomines in Texas dog kennel environments: Triatomine collections, infection with Trypanosoma cruzi, and blood feeding hosts. Acta Trop. 250, 107087 (2024). 38061614 10.1016/j.actatropica.2023.107087

[37] Ludt W. B. & Rocha L. A. Shifting seas: the impacts of Pleistocene sea-level fluctuations on the evolution of tropical marine taxa. J. Biogeogr. 42, 25–38 (2015).

[38] Tollis M. & Boissinot S. Genetic variation in the green anole lizard (Anolis carolinensis) reveals island refugia and a fragmented Florida during the quaternary. Genetica 142, 59–72 (2014). 24379168 10.1007/s10709-013-9754-1 PMC4778398

[39] Oliveira J. Combined phylogenetic and morphometric information to delimit and unify the Triatoma brasiliensis species complex and the Brasiliensis subcomplex. Acta Trop. 170, 140–148 (2017). 28219669 10.1016/j.actatropica.2017.02.020 PMC8259052

[40] Dujardin J.-P. The Body of Chagas Disease Vectors. Pathogens 14, 98 (2025). 39861059 10.3390/pathogens14010098 PMC11768379

[41] Dujardin J.-P., Beard C. B. & Ryckman R. The relevance of wing geometry in entomological surveillance of Triatominae, vectors of Chagas disease. Infect. Genet. Evol. 7, 161–167 (2007). 16949351 10.1016/j.meegid.2006.07.005

[42] Paschoaletto L. Morphological Stasis in Time? A Triatoma brasiliensis brasiliensis Study Using Geometric Morphometrics in the Long Run. Animals 12, 1362 (2022). 35681826 10.3390/ani12111362 PMC9179344

[43] Marquina D., Buczek M., Ronquist F. & Łukasik P. The effect of ethanol concentration on the morphological and molecular preservation of insects for biodiversity studies. PeerJ 9, e10799 (2021). 33614282 10.7717/peerj.10799 PMC7883690

[44] Killets K. C. Comparative Feeding and Defecation Behaviors of Trypanosoma cruzi-Infected and Uninfected Triatomines (Hemiptera: Reduviidae) from the Americas. Insects 16, 188 (2025). 40003818 10.3390/insects16020188 PMC11856564

[45] Pippin W. F. The Biology and Vector Capability of Triatoma Sanguisuga Texana Usinger and Triatoma Gerstaeckeri (StÅL) Compared With Rhodnius Prolixus (StÅL) (Hemiptera: Triatominae)1. J. Med. Entomol. 7, 30–45 (1970). 4907991 10.1093/jmedent/7.1.30

[46] Zeledón R., Alvarado R. & Jirón L. F. Observations on the feeding and defecation patterns of three triatomine species (Hemiptera: Reduviidae). Acta Trop. 34, 65–77 (1977). 16468

[47] Curtis-Robles R., Wozniak E. J., Auckland L. D., Hamer G. L. & Hamer S. A. Combining Public Health Education and Disease Ecology Research: Using Citizen Science to Assess Chagas Disease Entomological Risk in Texas. PLoS Negl. Trop. Dis. 9, e0004235 (2015). 26658425 10.1371/journal.pntd.0004235 PMC4687635

[48] Monteiro F. A. Molecular phylogeography of the Amazonian Chagas disease vectors Rhodnius prolixus and R. robustus. Mol. Ecol. 12, 997–1006 (2003). 12753218 10.1046/j.1365-294x.2003.01802.x

[49] Geospiza I. FinchTV 1.4.0. (2009).

[50] Sayers E. W. GenBank. Nucleic Acids Res. gkz956 (2019) doi:10.1093/nar/gkz956.

[51] Katoh K. & Standley D. M. MAFFT multiple sequence alignment software version 7: improvements in performance and usability. Mol. Biol. Evol. 30, 772–780 (2013). 23329690 10.1093/molbev/mst010 PMC3603318

[52] Paradis E. & Schliep K. ape 5.0: an environment for modern phylogenetics and evolutionary analyses in R. Bioinformatics 35, 526–528 (2019). 30016406 10.1093/bioinformatics/bty633

[53] Paradis E. pegas: an R package for population genetics with an integrated-modular approach. Bioinformatics 26, 419–420 (2010). 20080509 10.1093/bioinformatics/btp696

[54] Cheng L., Connor T. R., Sirén J., Aanensen D. M. & Corander J. Hierarchical and spatially explicit clustering of DNA sequences with BAPS software. Mol. Biol. Evol. 30, 1224–1228 (2013). 23408797 10.1093/molbev/mst028 PMC3670731

[55] Puillandre N., Lambert A., Brouillet S. & Achaz G. ABGD, Automatic Barcode Gap Discovery for primary species delimitation. Mol. Ecol. 21, 1864–1877 (2012). 21883587 10.1111/j.1365-294X.2011.05239.x

[56] Kapli P. Multi-rate Poisson tree processes for single-locus species delimitation under maximum likelihood and Markov chain Monte Carlo. Bioinformatics 33, 1630–1638 (2017). 28108445 10.1093/bioinformatics/btx025 PMC5447239

[57] Pons J. Sequence-Based Species Delimitation for the DNA Taxonomy of Undescribed Insects. Syst. Biol. 55, 595–609 (2006). 16967577 10.1080/10635150600852011

[58] Ezard T., Fujisawa T. & Barraclough T. splits: SPecies’ LImits by Threshold Statistics. R package version 1.0-14/r31. (2009).

[59] Suchard M. A. Bayesian phylogenetic and phylodynamic data integration using BEAST 1.10. Virus Evol. 4, (2018). 10.1093/ve/vey016 PMC6007674 29942656

[60] Rambaut A., Drummond A. J., Xie D., Baele G. & Suchard M. A. Posterior Summarization in Bayesian Phylogenetics Using Tracer 1.7. Syst. Biol. 67, 901–904 (2018). 29718447 10.1093/sysbio/syy032 PMC6101584

[61] Nguyen L.-T., Schmidt H. A., Von Haeseler A. & Minh B. Q. IQ-TREE: A Fast and Effective Stochastic Algorithm for Estimating Maximum-Likelihood Phylogenies. Mol. Biol. Evol. 32, 268–274 (2015). 25371430 10.1093/molbev/msu300 PMC4271533

[62] Schliep K. P. phangorn: phylogenetic analysis in R. Bioinformatics 27, 592–593 (2011). 21169378 10.1093/bioinformatics/btq706 PMC3035803

[63] Yu G., Smith D. K., Zhu H., Guan Y. & Lam T. T. ggtree : an R package for visualization and annotation of phylogenetic trees with their covariates and other associated data. Methods Ecol. Evol. 8, 28–36 (2017).

[64] Wickham H. ggplot2. WIREs Comput. Stat. 3, 180–185 (2011).

[65] Leigh J. W. & Bryant D. popart : full-feature software for haplotype network construction. Methods Ecol. Evol. 6, 1110–1116 (2015).

[66] Wickham H., François R., Henry L., Müller K. & Vaughan D. dplyr: A Grammar of Data Manipulation. 1.2.0 10.32614/CRAN.package.dplyr (2014).

[67] Wickham H. tidyr: Tidy Messy Data. R package version 1.3.1. WIREs Comput. Stat. https://github.com/tidyverse/tidyr (2024).

[68] Becker R. A., Wilks A. R., Brownrigg R., Minka T. P. & Deckmyn A. maps: Draw Geographical Maps. 3.4.3 10.32614/CRAN.package.maps (2003).

[69] Yu G. scatterpie: Scatter Pie Plot. 0.2.6 10.32614/CRAN.package.scatterpie (2016).

[70] Wishart D. S. & Fortin S. The BioTools Suite. A comprehensive suite of platform-independent bioinformatics tools. Mol. Biotechnol. 19, 59–77 (2001). 11697221 10.1385/MB:19:1:059

[71] Venables W. N. & Ripley B. D. Modern Applied Statistics with S. (Springer, New York, 2002).

